# Distinct Human Gut Microbial Taxonomic Signatures Uncovered With Different Sample Processing and Microbial Cell Disruption Methods for Metaproteomic Analysis

**DOI:** 10.3389/fmicb.2021.618566

**Published:** 2021-07-05

**Authors:** Carmen García-Durán, Raquel Martínez-López, Inés Zapico, Enrique Pérez, Eduardo Romeu, Javier Arroyo, María Luisa Hernáez, Aida Pitarch, Lucía Monteoliva, Concha Gil

**Affiliations:** ^1^Departamento de Microbiología y Parasitología, Facultad de Farmacia, Universidad Complutense de Madrid, Madrid, Spain; ^2^Unidad de Proteómica, Facultad de Farmacia, Universidad Complutense de Madrid, Madrid, Spain

**Keywords:** metaproteomics, gut microbiota, sample preparation, cell disruption, human proteins, taxonomic profiles

## Abstract

The use of metaproteomics for studying the human gut microbiota can shed light on the taxonomic profile and the functional role of the microbial community. Nevertheless, methods for extracting proteins from stool samples continue to evolve, in the pursuit of optimal protocols for moistening and dispersing the stool sample and for disrupting microbial cells, which are two critical steps for ensuring good protein recovery. Here, we evaluated different stool sample processing (SSP) and microbial cell disruption methods (CDMs). The combination of a longer disintegration period of the stool sample in a tube rotator with sonication increased the overall number of identified peptides and proteins. Proteobacteria, Bacteroidetes, Planctomycetes, and Euryarchaeota identification was favored by mechanical cell disruption with glass beads. In contrast, the relative abundance of Firmicutes, Actinobacteria, and Fusobacteria was improved when sonication was performed before bead beating. Tenericutes and Apicomplexa identification was enhanced by moistening the stool samples during processing and by disrupting cells with medium-sized glass beads combined with or without sonication. Human protein identifications were affected by sonication. To test the reproducibility of these gut metaproteomic analyses, we examined samples from six healthy individuals using a protocol that had shown a good taxonomic diversity and identification of proteins from Proteobacteria and humans. We also detected proteins involved in microbial functions relevant to the host and related mostly to specific taxa, such as B12 biosynthesis and short chain fatty acid (SCFA) production carried out mainly by members in the *Prevotella* genus and the Firmicutes phylum, respectively. The taxonomic and functional profiles obtained with the different protocols described in this work provides the researcher with valuable information when choosing the most adequate protocol for the study of certain pathologies under suspicion of being related to a specific taxon from the gut microbiota.

## Introduction

The human gut microbiota is a complex community of microorganisms that inhabit the human gastrointestinal tract. The gut microbiota comprises nearly a thousand bacterial species, in addition to archaea, fungi, parasites, and viruses (Alarcón et al., 2016; Lloyd-Price et al., 2016). When this ecosystem is balanced and shows high diversity, its close relationship with the host provides beneficial effects, such as the digestion of indigestible foods, defense against pathogens, immunomodulation, and production of vitamins and other beneficial products (Jandhyala et al., 2015). However, even with its great capacity for resilience, the gut microbiota is influenced by many factors, including diet, age, pollution, and the consumption of antibiotics, among others (Jakobsson et al., 2010; David et al., 2014; Jin et al., 2017; Kim and Jazwinski, 2018; Adak and Khan, 2019). These factors can affect its composition and biodiversity, leading in some cases to dysbiosis. Although it remains unclear whether dysbiosis is a cause or consequence of host pathological states, the link between pathological states and gut microbiota dysbiosis is a proven fact. Gut microbiota dysbiosis was previously associated with several disease states, including gastrointestinal diseases (Ni et al., 2017; Saffouri et al., 2019), cancer (Wang et al., 2019), Alzheimer’s disease (Kowalski and Mulak, 2019), and even autism (Fattorusso et al., 2019). Furthermore, the microbiota-gut-brain axis is the link between the gut microbiota and host pathologies related to mental conditions (Wang and Wang, 2016). This link illustrates the importance of the metabolic pathways carried out by gut microbiota, which produce a high diversity of enzymes and metabolites that perform several functions in different parts of the body, not exclusively in the gastrointestinal tract.

Currently, metagenomics is the most common “omic” approach for studying the microbiota, due to its ability to provide valuable information about microbial complexity (Jovel et al., 2016). Nevertheless, this strategy lacks the ability to provide functional insights. In this context, metaproteomics is a promising approach for studying the gut microbiota, both from the taxonomic point of view and the functional point of view. Indeed, metaproteomics can reveal the main metabolic and functional roles played by the different microorganisms present in gut microbiota (Zhang et al., 2016; Issa Isaac et al., 2019). Moreover, this approach can provide information about proteins that mediate the interactions between the gut microbiota and the host (Blackburn and Martens, 2016). Thus, metaproteomics can provide a better understanding of the functional roles of the gut microbiota, compared to other “omics” approaches (Zhang et al., 2019).

Despite advancements in metaproteomic techniques in recent years (Zhang and Figeys, 2019), the complexity of the microbiota samples, typically derived from stool samples, has made it difficult to devise a suitable protocol for maximizing microorganism recovery. First, microbial cells must be separated and extracted from the fecal sample. Regarding this issue, differential centrifugation has been found to provide good results in the enrichment of the bacterial fraction (>90%), revealing a low microbial cell retention by other fecal components (Apajalahti et al., 1998). Tanca et al. (2015) also evaluated the effectiveness of this process. In both works, and prior to this differential centrifugation, the humectation of the fecal sample in a tube rotator for several minutes was carried out. In contrast, Zhang et al. (2018) substituted this several minutes tube rotator humectation and dispersing step for vortexing the fecal sample with large-sized glass beads saving time in the sample processing. In addition, an optimal microbial cell disruption method (CDM) is needed to ensure that protein identification is as comprehensive as possible. Because protein extraction methods can affect metaproteomics results (Zhang et al., 2018), the protocol must be optimized to produce representative taxonomic profiles and identify the most significant metabolic roles carried out by gut microbiota. Regarding microbial cell disruption, several techniques have been used in microbiota studies by different authors using different lysis buffers and different mechanical disruption methods. Concerning lysis buffers, these studies pointed out SDS-containing buffer to be the one that rendered the best results in protein yields in comparison to other buffers tested (Zhang et al., 2018). Regarding the mechanical disruption, bead-beating and sonication are the two main options for this purpose and have been widely used in different metaproteomics studies (Santiago et al., 2014; Tanca et al., 2014; Zhang et al., 2016). As for bead-beating, the suitable size of the beads to be used in gut microbiota studies is not well defined yet. Small beads (<0.5 mm) (Wu et al., 2016; Zhang et al., 2018; Schultz et al., 2020) have been normally used in this type of studies, while bigger glass beads (>0.5 mm) are normally used for yeast cell disruption (Pitarch et al., 2008). A mixture of different sized beads has been proven to better extract proteins from gram-positive bacteria and yeast (Hayoun et al., 2019). We therefore wanted to test several human gut protein extraction approaches using different sized-beads in combination or not with an additional sonication step prior to bead-beating.

In addition to optimizing the extraction of microbial proteins from a stool sample, bioinformatics is a crucial step in the metaproteomics workflow. Different software for peptides identifications are available, like MaxQuant (Cox and Mann, 2008) or X! TANDEM (Craig and Beavis, 2004). Moreover, there are several tools that allow the functional characterization of the identified peptides (Sajulga et al., 2020). There are also different options available that combine both, peptides identification and functional annotation, like the MetaProteomeAnalyzer (Muth et al., 2015), Unipept (Mesuere et al., 2016), or MetaLab (Cheng et al., 2017). These software are open-source and easy-to-use tools. The use of these software has facilitated the analysis of the huge amount of data generated in metaproteomics studies of human gut microbiota. We have used the software MetaLab for the bioinformatics analysis carried out in this work. This software accesses a protein dataset that was created from more than 1,000 human microbiota samples, which allows a high number of peptide/protein identifications. As explained before, the software also allows functional characteristics to be assigned to the identified proteins at specific taxon levels.

In this study, we aimed to compare the isolation and characterization of human gut microbiota by analyzing one stool sample with different protocols combining different sample processing and microbial CDMs. We assessed the effectiveness of one of these assayed protocols by characterizing and comparing six human gut microbiome samples, according to their taxon profiles and the functions of the identified proteins in each individual sample.

## Materials and Methods

### Stool Samples

To test the different protocols, a stool sample from a healthy volunteer (from now on named H1) was used. To perform the metaproteomic study, stool samples were collected from 6 healthy adult volunteers with their informed consent. The 6 samples belonged to 3 females and 3 males, aged between 31 and 52 and were named, S1 to S6. None of them had been under antibiotics treatment during the previous year to sampling. Only one subject reported gastrointestinal problems within 3 months prior to sampling. Nutritional habits of the volunteers are unknown. Feces were stored at −80°C until processing.

### Metaproteomic Analysis of Stool Samples

**FIGURE 1:**
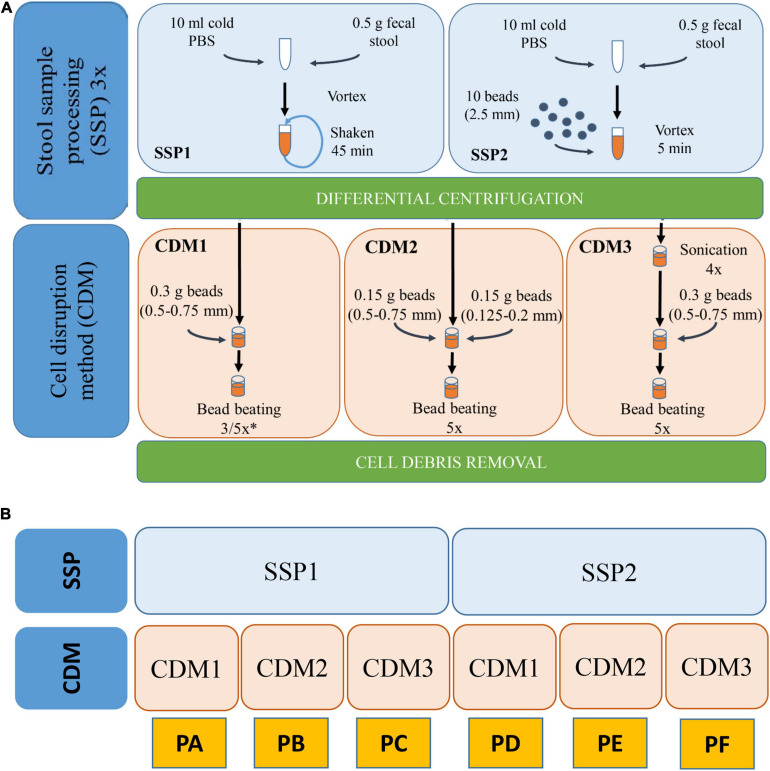
**(A)** Workflow of the various SSP (stool sample processing) and CDM (cell disruption method) carried out with stool sample H1. **(B)** Schematic representation of the different combinations of SSP and CDM carried out in each protocol (PA–PF). ^∗^In CDM1, 3 rounds were performed for PA and 5 rounds for PD.

We combined two different stool sample processing (SSP) and three CDMs. All these protocols are schematized in Figure 1. Each protocol was performed in triplicate.

#### Microbial Enrichment by SSP

SSP1 was modified from different works (Tanca et al., 2015; Zhang et al., 2018). Briefly, 0.5 g of the stool sample were resuspended in 10 mL ice-cold phosphate buffer (PBS), vortexed and shaken in a tube rotator for 45 min at 4°C. The hydrated samples were subsequently subjected to low-speed centrifugation at 500 × *g*, 4°C for 5 min which favored the sedimentation of particulate and insoluble material allowing the collection of the supernatant (containing microbial cells). The supernatant was then transferred to a new tube and kept at 4°C while the pellets were resuspended in 10 mL ice-cold PBS, repeating this process two other times. Finally, the supernatants (∼30 mL) were centrifuged at 14,000 × *g* and 4°C for 20 min to pellet the microbial cells. Pellet was then washed three times with PBS. The washing step was performed by resuspension of cell microbial pellet in ice-cold PBS followed by centrifugation at 14,000 × *g* and 4°C for 20 min. The resulting washed pellet was subjected to protein extraction. SSP2 is a variation of SSP1 in which the 45 min shaking step in the tube rotator was changed for a 5 min vortexing step with 10 glass beads (2.5 mm).

#### Cell Disruption Method for Protein Extraction

Three different CDMs (1–3) were carried out for cell disruption and protein extraction. CDM1 was modified from Zhang et al. (2018). Briefly, the microbial pellet was resuspended in 500 μL lysis buffer (4% SDS (w/v) in 50 mM Tris–HCl buffer pH 8.0) followed by incubation in a Thermomixer Comfort (Eppendorf) at 95°C for 10 min with agitation. After cooling, the lysates were transferred to a 2-mL screw-cap tube containing 0.3 g glass beads (0.5–0.75 mm) (Retsch). Bead beating was carried out using a FastPrep-24 machine (MP Biomedicals Inc., United States) at a speed of 6.5 ms^–1^ for 90 s (3 rounds 30 s each with 5 min interval on ice) in CMD1 for protocol A or for 150 s (5 rounds 30 s each with 5 min interval on ice) for protocol D. Finally, cell debris and beads were removed through centrifugation at 16,000 × *g* and 4°C for 10 min. The supernatant was then transferred into a new tube and centrifuged again at 16,000 × *g* and 4°C for 10 min to remove any remaining particulate debris. The final supernatant was used for protein quantification and purification. CDM2 was performed similarly to CDM1 but introducing two different sizes of glass beads (0.15 g of 0.5–0.75 mm and 0.15 g of 0.125–0.2 mm). CDM3 was modified from Zhang et al. (2017). Briefly, the microbial pellet was resuspended in a 500 μL volume of 4% SDS (w/v) lysis buffer followed by incubation in a Thermomixer Comfort at 95°C for 10 min with agitation. After cooling and once the lysates had been transferred to a 2-mL screwcap tube, 4 sonications (30 s each with 1 min interval on ice) using Vibra Cell Sonicator (Sonics & Materials Inc., United States) with an amplitude of 40% were carried out. After sonication, bead beating disruption was carried out under conditions (including beads sizes, speed and rounds) described in CDM1. Combination of the two different SSPs and three CDMs resulted in six protocols named A–F (Figure 1).

### Peptide Sample Preparation for Mass Spectrometry

Proteins were precipitated using methanol/chloroform to remove SDS, and then resuspended in 8 M urea for in-solution trypsin digestion. The samples were quantified with Qubit 3.0 (Thermo Fisher Scientific) and 15 μg of each sample were loaded and separated on an SDS-PAGE gel to confirm protein concentration and protein pattern to check the sample suitability for metaproteomic study. Subsequently, 50 μg of proteins were reduced with 10 mM dithiothreitol (DTT) at 37°C for 45 min and alkylated with 55 mM iodoacetamide (IAA) at room temperature for 30 min in darkness. 1:25 trypsin (enzyme:protein) (Roche Molecular Biochemicals) was added to each sample in 25 mM ammonium bicarbonate for trypsin digestion at 37°C overnight. The peptides from protein digestions were desalted and concentrated with C18 reverse phase chromatography (OMIX C18, Agilent technologies) being later eluted with 80% acetonitrile (ACN)/0.1% trifluoroacetic acid. The eluent was then freeze-dried in Speed-vac (Savant) and resuspended in 12 μL 2% ACN/0.1% formic acid (FA). Finally, peptides were quantified in Qubit fluorimeter (Thermo Fisher Scientific) and 1 μg peptides were used for liquid chromatography (LC)-mass spectrometry (MS)/MS analysis.

### Liquid Chromatography-Tandem Mass Spectrometry Analysis

For RP-nano-LC-ESI-MS/MS analysis, 1 μg peptides were analyzed in a Q Exactive HF mass spectrometer coupled to an EASY – nLC 1000 System (Thermo Fisher Scientific).

First, the peptides were loaded on-line onto a Acclaim PepMap 100 Trapping column (75 μm i.d. × 20 mm, 3 μm C18 resin with 100 Å pore; Thermo Fisher Scientific) using buffer A (0.1% FA) and then separated on a C18 resin analytical column (75 μm i.d. × 500 mm, 2 μm and 100 Å pore size; Thermo Scientific Easy Spray Column) with an integrated spray tip. The flow rate during the sample loading state was 250 nL/min. The peptides were separated by a binary buffer system of 0.1% FA (buffer A) and 0.1% FA in 100% ACN (buffer B). A 240 min gradient from 2 to 40% buffer B in buffer A was performed.

Data were acquired in a Full-MS data-dependent acquisition (DDA) in a positive mode with Xcalibur 4.1 software in a Q-Exactive HF hybrid quadrupole-Orbitrap mass spectrometer (Thermo Fisher Scientific). MS scans were acquired at m/z range of 350 to 2,000 Da followed by data-dependent MS/MS scan (with a threshold of 0.01) of the 15 most abundance precursors with charges of 2–5 in MS scans for high-energy collision dissociation (HCD) fragmentation with a dynamic exclusion of 10 s and normalized collision energy (NCE) of 20 and the corresponding MS/MS spectra were acquired.

The mass spectrometry proteomics raw data have been deposited to the ProteomeXchange Consortium^1^ via PRIDE partner repository with the database identifiers PXD020786 and PXD025659.

### Bioinformatics Analysis

For data processing, MetaLab version 2.0 was used (Cheng et al., 2017). This software provides peptide and protein identification and quantification, taxonomic profiling and functional annotation. Briefly, the human gut microbial gene catalog with 9,878,647 sequences^2^ is employed for generating a reduced, sample-specific protein database (Li et al., 2014). The resulting database is combined with a human database in order to also identify human proteins. This human database was downloaded from Uniprot DB^3^ (UniProt Consortium, 2019) restricted to human taxonomy (downloaded 02.18.2020 with 74,451 sequences). This combined database was used for peptide characterization using the Andromeda search engine from MaxQuant version 1.6.10.43 which is integrated in MetaLab. Quantitative analysis is performed employing the maxLFQ algorithm in MaxQuant. Proteins identified by the same set or a subset of peptides are grouped together as one protein group. For protein identification MetaLab software requires the detection of one exclusive peptide thereof. The FDR is set as 0.01. Taxonomic analysis of the generated peptide list is performed matching peptides to the lowest common ancestor (LCA). Abundance data of both peptides and taxa were provided according to the LFQ intensities. The quantitative information of each taxon was calculated summing up all the intensities belonging to all distinctive peptides assigned to that taxon. For each taxon, this value was then used to estimate its relative abundance within all the taxa identified in the sample. The taxonomic results were manually filtered to not retain human taxa and only taxa identified with at least three peptides were considered.

Functional annotation for the identified proteins was also assigned and automated generated from MetaLab to directly obtain information about clusters of orthologous groups (COGs) and kyoto encyclopedia of genes and genomes (KEGG). For deep functional analysis, KEGG Orthology (KO) database information (molecular functions as functional orthologs defined in the context of KEGG molecular networks) was used (see text footnote 2). Taxon-function analysis was carried out using taxonomic information of the enrichment analysis from iMetaLab platform^4^ in combination with KO database information.

KEGG pathway was downloaded from KEGG website^5^. STRING analysis was done with the STRING program (version 11.0). Graphs were made with Infogram^6^.

### Statistical Analysis

The Kolmogorov–Smirnov test and Shapiro–Wilk test were applied to examine whether data followed a normal distribution. The independent-samples Student’s *t*-test or Mann–Whitney *U* test was used to evaluate the central tendencies of numeric variables between two categorical independent groups, as appropriate. One-way analysis of variance (ANOVA) with *post hoc* Tukey’s test was applied to compare these tendencies among multiple independent groups. Unsupervised principal component analysis (PCA) was carried out to group the stool samples from one selected healthy individual (H1) extracted with the different established protein extraction protocols on the basis of the similarities in their gut microbial taxonomic abundance patterns observed from the metaproteomic data. The degree of homology or relative similarity of these profiles among the study groups was assessed with Mann–Whitney *U* test, whereas their degree of homogeneity or relative variation within each study group was evaluated by ANOVA. Unsupervised two-way hierarchical clustering analysis (HCA) was performed to cluster the stool samples extracted with the six protocols and different identified (phylum, family, or genus) taxa simultaneously according to the similarities in the gut microbial taxonomic abundance profiles of each extraction method and the pattern of each identified gut microbial taxon across all protein extraction protocols, respectively. Relative abundance rates were normalized by median centering the gut microbial taxonomic abundance profiles for each protein extraction protocol and then by median centering each abundance pattern of each identified microbial taxon across the six protocols. Statistical analyses were carried out using the GraphPad Prism and IBM SPSS Statistics programs, as well as the Python SciPy and Seaborn packages. Statistical significance was set at *p* < 0.05 (two-sided).

## Results and Discussion

### Protein Extraction Protocol Effects on Protein Yield and Identification

In metaproteomics, protein identification yield depends on the method used for protein isolation and solubilization, particularly in complex samples, such as stool samples (Zhang et al., 2018). We used one stool sample from a healthy volunteer (H1) to examine the effects of six different protein extraction protocols (referred to as PA, PB, PC, PD, PE, and PF) for the metaproteomic analysis of gut microbiota (Figure 1). These protocols included two main steps: (i) SSP, which is needed to disperse the feces to harvest the microbial cells, and (ii) a CDM for breaking up microbial cells to isolate and solubilize their proteins. In PA, PB and PC, the SSP was performed with three rounds of 45-min shaking/low speed centrifugation (SSP1). In PD, PE, and PF, a faster, easier SSP was devised by adding big glass beads and vortexing for 5 min (SSP2). We also compared different CDMs by testing different sizes of smaller glass beads (PB and PE) and an additional sonication step (PC and PF).

**FIGURE 2:**
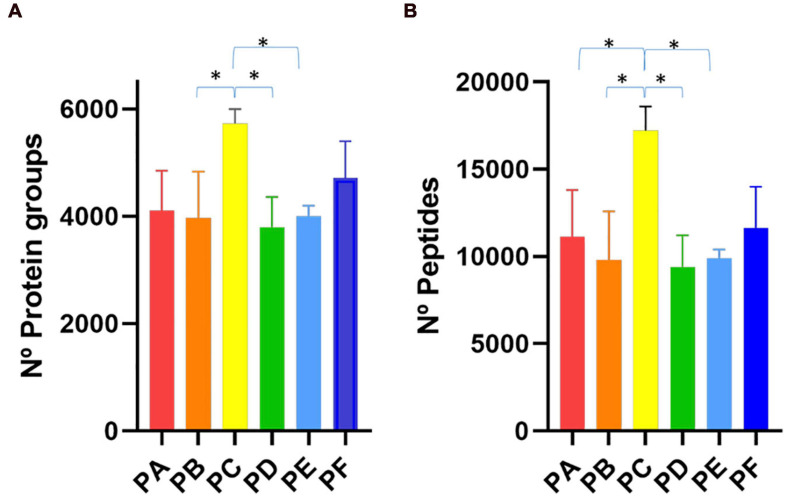
Bar plot representing the number of **(A)** protein groups and **(B)** peptides identifications achieved with the six different protocols tested (PA–PF). ^∗^*p* < 0.05.

First, we compared PA, PB and PC with protocols PD, PE and PF which differed in the SSP (Figure 1). We observed that the first group of protocols provided a higher number of peptide-spectrum matches (PSMs) (Supplementary Table 1) and more peptide and protein identifications than the latter ones (Figure 2 and Supplementary Table 1). These results indicated that the longer treatment carried out in SSP1 with three 45-min shaking rounds was important to disintegrate the stool sample correctly, allowing the recovery of as many microbial cells as possible. To test the effect of the use of beads with different sizes, we compared PA with PB and PD with PE. In the group processed with the SSP1 (PA and PB) the addition of two different sized beads seemed to negatively influence the protein and peptides identification yield while in the second group (PD and PE) opposite trend was observed (Figure 2). Nevertheless, there were no significant differences in the number of identified proteins and peptides depending on whether a combination of beads was used (PA vs. PB or PD vs. PE, Figure 2). The most drastic effect in protein recovery from the gut microbiota, reflected in the number of PSMs, peptide and protein group identifications, was due to the addition of sonication in the CDM (Figure 2). The increase in the number of peptide and protein identifications obtained when using sonication reached statistical significance when compared to protocols where cell disruption was carried out exclusively by bead beating. Furthermore, PC that uses 45 min shaking (SSP1) and sonication in the CDM step showed the highest number of peptide and protein identifications within all protocols, but with the disadvantage of being a more consuming process due to the additional steps.

### Impact of Protein Extraction Methods on Taxonomic and Functional Profiles of Human Gut Microbiota

Next, we explored the influence of the six protein extraction protocols on the ability to identify the taxonomic composition of the gut metaproteome in H1. It is worth mentioning that unlike taxonomic metagenomic studies, which are based on 16S rRNA gene sequencing, metaproteomic analyses allow the identification of human proteins. The identification of these proteins could be advantageous in some dysbiosis-related pathological conditions studies, mainly when the pathological condition includes inflammation of the gastrointestinal tract since inflammation related proteins could be tracked to check the patient’s evolution. Interestingly, the detection of human peptides was diminished in PC and PF (Supplementary Figure 1) thus sonication seems to be unfavorable for human proteins identification. Nevertheless, for microbial taxonomic purposes, we filtered out the human proteins and focused exclusively on non-human peptides.

**FIGURE 3:**
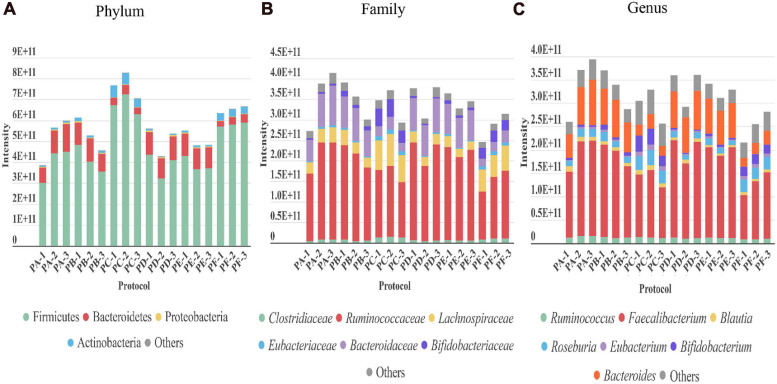
Taxonomic distribution revealed by each of the three replicates performed with every protocol (PA–PF) from stool sample H1. The intensity of each taxon in the different taxonomic levels **(A)** phylum, **(B)** family, and **(C)** genus (directly related to its abundance) is represented as the sum of the intensities of all the distinctive peptides assigned to that taxon.

Regarding the gut microbiota, the taxonomic profile at the phylum, family, and genus levels revealed that the presence of the main phyla, families, and genera displayed by the different protocols were in general comparable among them (Figure 3). However, PC and PF (including sonication) showed several differences. They achieved a higher peptide and protein identifications (Figure 2). These protocols also showed a greater level of identification in the higher taxonomic levels (superkingdom and phylum) (Figure 3 and Supplementary Figures 1, 2). Nevertheless, the intensity of the peptides that were assigned to lower taxonomic levels such as family and genus were very similar or even slightly lower in protocols including sonication (Figure 3 and Supplementary Figure 2). Regarding taxa distribution, we also observed some clear differences in PC and PF in comparison to the others such as an increased proportion of Actinobacteria or a decreased abundance in Bacteroidetes.

**FIGURE 4:**
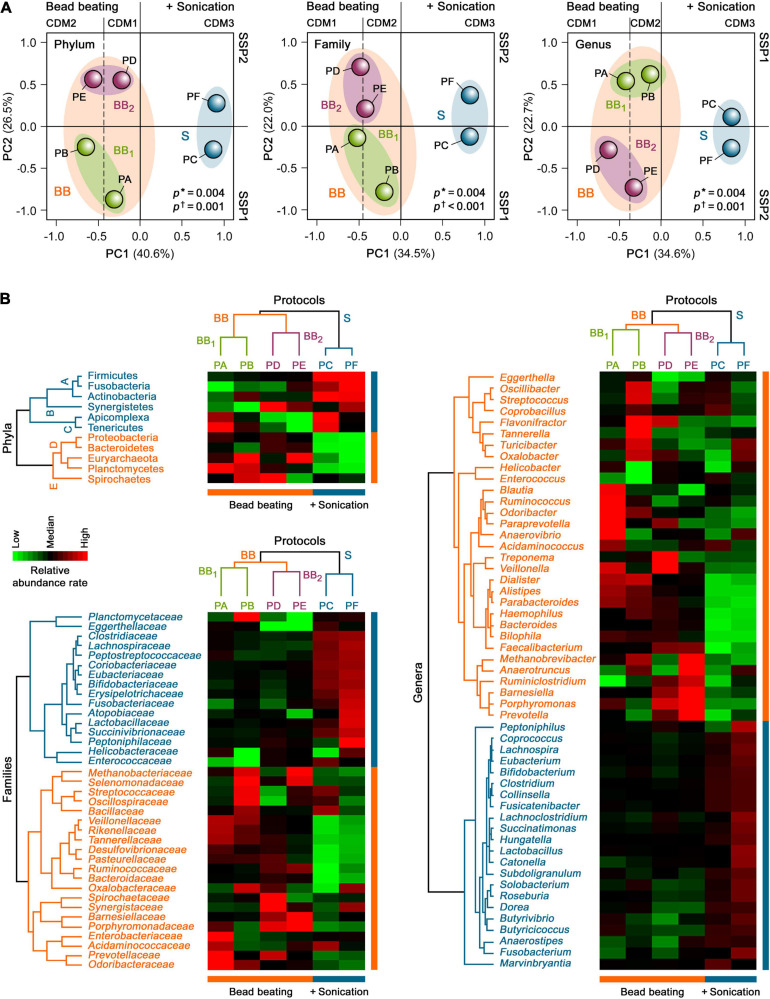
Unsupervised **(A)** PCA and **(B)** HCA of the taxonomic profiles of the gut metaproteome of H1 isolated with different microbial protein extraction protocols. *Asterisks* show the degree of homology of taxonomic abundance profiles of human gut microbiota of H1 between the study groups, whereas *daggers* indicate their degree of homogeneity within each study group.

To further examine the global taxonomic profiles of human gut microbiota derived from each protein extraction protocol in H1 as a whole, an unsupervised PCA was performed on the relative mean taxonomic abundance rates at the phylum, family and genus levels. PCA revealed that the taxonomic abundance patterns obtained with CDM based on bead beating were distinct from those that included an additional sonication step before bead beating (clusters BB and S; *p* = 0.004; Figure 4A). PCA further highlighted that their individual variances also differed between both groups (*p* ≤ 0.001), indicating that these profiles were heterogeneous within these two CDM groups. Remarkably, 34.5–40.6% of the total variance of taxonomic compositions of the gut metaproteome at the phylum, family and genus levels in H1 was attributed to differences in CDM (i.e., among CDM1, CDM2, and CDM3), while 22.0–26.5% of the dataset variance was explained by dissimilarities in SSP (i.e., between SSP1 and SSP2), as shown by the two first principal components (PC1 and PC2, respectively). These data suggested that CDM had a markedly higher impact on the taxonomic compositional changes in the human gut metaproteome than SSP.

Consistent with the PCA results, an unsupervised two-way HCA unveiled two major clusters of highly correlated taxonomic abundance profiles of the gut metaproteome in H1 that differentiated between CDM based on only bead beating (PA, PB, PD, and PE) and sonication plus bead beating (PC and PF) at the phylum, family, and genus levels (clusters BB and S; Figure 4B). In turn, cluster BB integrated two subclusters of more strongly correlated taxonomic abundance patterns that segregated bead beating-based CDM in two groups, BB1 and BB2, related to SSP1 (PA and PB) and SSP2 (PD and PE), respectively.

Hierarchical clustering analysis further uncovered specific gut microbial taxonomic signatures at the phylum level that were associated with the different SSP and CDM used for the six protein extraction protocols (Figure 4B). The CDM3-associated taxonomic signature (subcluster A) included the bacterial phyla, Firmicutes, Actinobacteria and Fusobacteria (*p* = 0.006). This finding showed the effect of an additional sonication step before mechanical cell disruption with medium-sized glass beads. Thus, PC and PF (CDM3) facilitated gut microbial protein extractions from Gram-positive bacteria (Firmicutes and Actinobacteria), which are more difficult to lyse than Gram-negative bacteria (Dridi et al., 2009; Salonen et al., 2010; Zhang et al., 2018). In addition, PC and PF enhanced protein extractions from Fusobacteria, which is a phylum of Gram-negative bacteria that has intriguingly been shown to share more traits with Gram-positive Firmicutes than with other Gram-negative bacteria (Robinson et al., 2020). The SSP2-associated taxonomic signature (subcluster B) comprised the bacterial phylum Synergistetes (*p* = 0.04). This result suggested that SSP based on beating with big-sized glasses (PD, PF, and to a lesser extent, PE) could facilitate protein extractions of this rare phylum from the human gut microbial community. In contrast, the taxonomic signature associated with SSP1, mostly with PA and PC (subcluster C), encompassed the bacterial phylum, Tenericutes, as well the unicellular eukaryotic parasite phylum, Apicomplexa (*p* = 0.02). These data highlighted that the gentle SSP1 (PA, PC, and to a lesser extent, PB) could be a crucial step for protein extractions of these low abundant phyla of microorganisms lacking cell walls (i.e., without mechanical support and strength) from the human gut microbiota.

The BB-associated taxonomic signature (subcluster D) comprised the Gram-negative bacterial phyla, Proteobacteria, Bacteroidetes, and Planctomycetes, and displayed a significantly higher proportion of the archaeal phylum, Euryarchaeota (*p* = 0.002). In particular CDM1 (PA and PD) enhanced protein extractions of Proteobacteria, supporting the notion that medium-sized glass beads for cell disruption could favor protein extractions from these Gram-negative bacteria in the human gut microbial community (*p* = 0.03). CDM2 (PB and PE) improved gut microbial protein extractions of Euryarchaeota (*p* = 0.02). These findings revealed that the combination of small and medium-sized glass beads added during intensive mechanical cell disruption could enhance protein extractions of hard-to-lyse gut microbiota members, such as methanogenic archaea and some Gram-positive bacteria, consistent with findings in previous studies (Dridi et al., 2009; Salonen et al., 2010; Zhang et al., 2018). BB1 (PA and PB) recovered a high proportion of Planctomycetes (*p* = 0.04), supporting the role of SSP1 on its protein extraction by bead beating from the gut microbial communities. In contrast, the PB and PD-associated taxonomic signature (subcluster E) integrated the bacterial phylum, Spirochaetes (*p* = 0.02). These data indicated that protein extractions of these Gram-negative bacteria, which have a unique cell envelope due to their endoflagella, from the human gut microbiota could be improved by gentle SSP followed by mechanical disruption with small and medium-sized glass beads (PB) or by the combination of SSP with big-sized glasses with CDM with medium-sized glass beads (PD).

Even though some works have avoided the preprocessing of the stool sample performed by differential centrifugation, carrying out a shortened and faster workflow using phenol extraction (Heyer et al., 2019) or adding directly the lysis buffer to the sample (Zhong et al., 2019), we have observed that this step provides an enrichment in diversity and a higher number of identifications, and it also reduces the information of non-microbial peptides, such as those derived from food remains that can affect the study (Tanca et al., 2015). Thus, we agree with other authors, that also observed a variation in the proportions of the microbial community with this preprocessing (Tanca et al., 2015), to consider this step relevant for this kind of studies even though it increases the whole workflow time (Zhang and Figeys, 2019). We have evaluated two different differential centrifugations, one comprising a faster 5-min vortexing method (SSP2) used in recent works (Zhang and Figeys, 2019) and another one using a more time-consuming process including a 45 min disintegrating process in a rotator tube (SSP1). Differences, both in protein and peptide identification as well as in taxonomics profiles, were observed. Moreover, both processing (SSP1 and SSP2) could be separated by PCA. SSP1 protocols showed, in general, higher peptide identifications. Furthermore, this slower processing was associated with a better identification of lower abundant microbiota taxa that are rarely observed in gut microbiota studies (Figure 4B).

Out of the three protocols using SSP1 (PA, PB, and PC), PC allowed a higher number of peptides and protein identifications with higher intensity in the identified peptides assigned to phylum level. However, regarding taxa distribution at family or genus levels, PA rendered better results providing also a greater number of identified human peptides relevant for the analysis of host-microbial interactions. The higher number of protein and peptide identifications using sonication had been previously described (Zhang et al., 2018), however, in our study, we have observed a reduction in peptide intensities assigned to low taxonomic levels. It had also been suggested in previous works that a mix of different sized beads could be a key factor for a good recovery of gut microbiota microorganisms (Hayoun et al., 2019), however, we did not observe statistical significance when this combination (CDM2) or just medium-sized beads (CDM1) were used (Figure 2). Nevertheless, we have found specific taxonomic signatures attached to this different CDM using bead beating, being Euryarchaeota related to CDM2, and Proteobacteria to CDM1 (Figure 4B).

Regarding time consuming of the different protocols, even if a more time consuming SSP1 is used, the overall time effort of the workflow is not greatly affected. To process one sample, nearly 48 h are required. Some studies reduce this period to a 24-h workflow, allowing the translation to clinics by means of a direct extraction of proteins from stool samples. This approach certainly shortens the time and can result in high benefit when it comes to detect specific proteins already defined as biomarkers of a particular disease (Heyer et al., 2019). Nevertheless, the human gut microbiome is among the most complex samples in terms not only of microorganisms but also in their diversity. Taking into account our results, to obtain a taxonomic profile from stool samples it is highly recommended a previous step of microbial enrichment, despite the increase of the time consumed.

In order to compare the functional profile obtained from the same sample with the six protocols carried out, the identified proteins were linked to their annotated function according to COG database. To assess the abundance of each function (COG category) we calculated the total intensity associated to that function by adding the intensity of every peptide assigned to it. The general functional profile was similar within all protocols, being able to detect the same COG categories, but the use of sonication in CDM3 (PC and PF) decreased the intensity of proteins lacking function assignment (blank) (*p* < 0.0001) while increasing the intensity of proteins assigned to major COG categories (those with the greater intensity) such as “translation, ribosomal structure and biogenesis” (J) (*p* < 0.0001), “carbohydrate transport and metabolism” (G) (*p* < 0.0001) and “energy production and conversion” (C) (PC *p* < 0.001, PF *p* < 0.015) (Supplementary Table 2).

### Metaproteomics Analysis of Gut Microbiota From Healthy Adults

We next carried out the metaproteomic analysis of gut microbiota from six healthy adults (S1–S6). For this analysis, we chose PA which includes the longer pre-treatment in a tube rotator already discussed to enhance microbial cells enrichment. Regarding CDM and protein extraction, even though PC showed a higher total number of peptides and protein identifications (Figure 2), this increase affected mainly at phylum level with a greater identification of Firmicutes, but without statistical difference at genus level compared to other protocols (Supplementary Figure 2). On the other hand, PA showed a higher abundance of Proteobacteria (Figures 3, 4) which is an important phylum in disease states, but their members are present in low abundance in the natural human gut microbiota. Moreover, Proteobacteria can serve as a potential diagnostic microbial signature of gut microbiota dysbiosis and disease risk (Shin et al., 2015). Finally, PA favored the detection of human proteins that could shed light on potential beneficial or detrimental relationships between the microbiota and the host (Supplementary Figure 1). We have summarized the main differences between PA and PC in Table 1.

**TABLE 1:** Summary table comparing PA and PC.

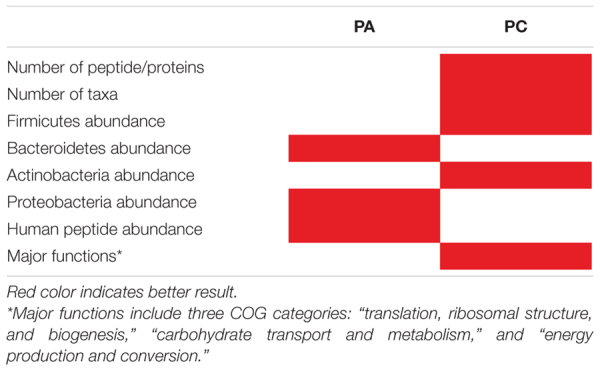

Upon characterizing microbial biodiversity, we focused on the contribution of each taxon to the metabolic processes and cell functions involved in the human gut environment, which could be linked to specific states of the host. In total, we identified 154,246 PSMs, 37,080 peptide sequences, and 10,686 protein groups. Among the 6 samples, the means were 25,503 PSMs, 11,712 peptide sequences, and 4,253 protein groups (Table 2). Considering only microbial proteins with at least 3 distinctive peptides, the identified proteins corresponded to 11 phyla, 19 classes, 25 orders, 34 families, 53 genera, and 105 species (Supplementary Table 3). We could not assign 17% of the identified peptides to any phylum. However, among all identified peptides, 27% could be assigned to species level. The percentage of peptides that could be assigned to superior taxon levels increased as the level increased: we assigned 54% at the genus level, 56% at the family level, 71% at the order level, and 72% at the class level.

**TABLE 2:** Metrics of metaproteome analysis of healthy adult stool samples (S1–S6).

	S1	S2	S3	S4	S5	S6	Mean
PSM identified	20,915	28,240	25,146	29,509	22,374	28,062	25,503
	154,246*						
PSM identified (%)	15	20	18	20	16	20	18
Peptide sequences	10,198	12,866	10,975	13,222	10,544	12,855	11,712
	37,080*						
Protein groups	3,926	4,602	3,732	4,525	4,110	4,722	4,253
	10,686*						

*Total number of PSM, peptides sequences or protein groups identified in the study.

#### Taxonomic Analysis of Gut Microbiota

**FIGURE 5:**
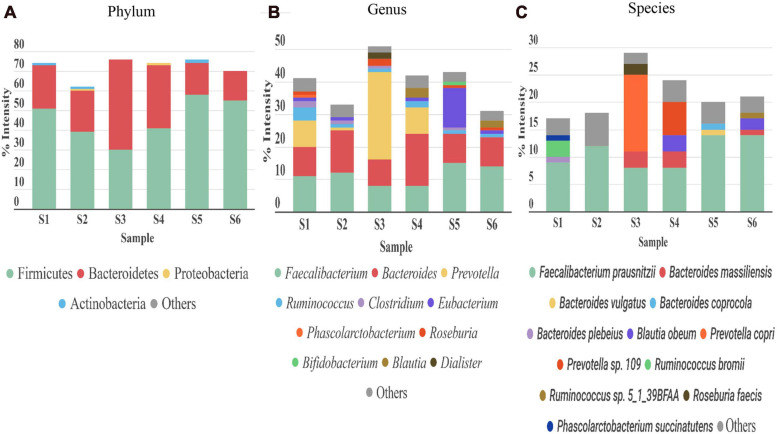
Taxonomic distribution of gut microbiota detected in stool samples from six healthy adults (S1–S6). **(A)** Phylum, **(B)** genus, and **(C)** species levels. Abundance of each phylum/genus/species is correlated to the percentage of peptide intensity ([sum intensity of peptides of the phylum]/[total intensity of sample]). Only those taxon categories with > 1% of abundance are represented in the graphs. The rest are included in the “other” category.

Bacteria was the most abundant superkingdom in the human gut microbiome; we found that it represented 96–99% of the microbiome, consistent with previous studies (Kolmeder et al., 2016; Tanca et al., 2017). In agreement with other works (Arumugam et al., 2011; Kolmeder et al., 2012, 2016; Tanca et al., 2017; Zhang et al., 2017; Zhong et al., 2019), the four most abundant phyla in our 6 samples were Firmicutes, Bacteroidetes, Proteobacteria, and Actinobacteria (Figure 5A). The dominant phylum was Firmicutes, except in S3, where Bacteroidetes was the principal phylum.

In the fungi kingdom, we found the Ascomycota phylum, but it was only present in 3 out of the 6 samples, and its mean abundance was 0.01% (Supplementary Table 3).

The most abundant genera were *Faecalibacterium* (phylum Firmicutes) and *Bacteroides* (phylum Bacteroidetes) (Figure 5B). *Ruminococcus*, another genus from Firmicutes, was highly abundant in S1 and S4. S1, S4, and particularly S3, were enriched in the genus *Prevotella* (phylum Bacteroidetes), which represented more than 8% in S1 and S4, and up to 27% in S3. A reciprocal pattern was reported for the abundances of *Prevotella* and *Bacteroides* (both from phylum Bacteroidetes) (Hoffmann et al., 2013), consistent with our results. The high abundance of *Prevotella* has been associated with a high consumption of fiber (David et al., 2014), and *Bacteroides* was linked to the Western diet, based on the relatively high meat consumption (Wu et al., 2011). Therefore, these differences in abundance among the samples could be partly explained by differences in diet among the corresponding individuals. In S3, we found *Akkermansia*, present at an abundance of 0.04% (Supplementary Table 3), consistent with a previous study (Kolmeder et al., 2012). A species of *Akkermansia* genus, *A. muciniphila*, is a mucin degrading bacteria. This species is gaining interest, because it induces several host responses, due to its proximity to the mucus layer. Indeed, *A. muciniphila* was associated with glucose control, inflammation, and improving the gut barrier (Everard et al., 2013). In the Actinobacteria phylum, *Bifidobacterium* was the main genus in our six samples; the average abundance was below 4%, consistent with previous studies (Riviere et al., 2016). Depending on the sample studied, we observed different compositions of taxa in the Proteobacteria phylum. This phylum is normally associated with disease states and gut microbiota dysbiosis (Rizzatti et al., 2017). The role of this phylum in abnormal physical states has been widely studied, but its contribution to normal conditions is less well known. The main genus in most of the samples was *Sutterella*. *Sutterella* spp. have been related to several gastrointestinal disorders, but it is thought that they only have a mild-proinflammatory capacity. However, they keep the immune system alert, due to their ability to adhere to intestinal epithelial cells (Hiippala et al., 2016). In this genus, the *Bilophila wadsworthia* was the only species which was identified in our study. This bacterium was associated with fat-rich diets and intestinal inflammation. Emerging studies have suggested that limiting the prevalence of *B. wadsworthia* in the gut microbiome could have a therapeutic effect on metabolic diseases and intestinal inflammation (Natividad et al., 2018).

At the species level (Figure 5C), consistent with previous studies (Kolmeder et al., 2012; Zhang et al., 2017; Lin et al., 2018), we found that the main species was *Faecalibacterium prausnitzii* (8–14%), except in S3, where *Prevotella copri* had an abundance of 14%. Previous studies showed that *F. prausnitzii* displayed beneficial effects against different alterations in the gastrointestinal tract (Bjørkhaug et al., 2019), and it had anti-inflammatory properties (Debyser et al., 2016). In the other hand, the role of *P. copri* in human health remains unclear. The presence of this species in the gut (more common in non-westernized populations) varies largely between individuals (Tett et al., 2019), but when it is present, it is normally the most abundant species (Arumugam et al., 2011), consistent with our findings.

#### Functional Characterization of Microbial Proteins

Focusing on a functional analysis, we used MetaLab to obtain information about the different functional assignments of the identified proteins (Cheng et al., 2017). In this metaproteomic approach, 89.5%, 64.7%, and 61.5% of the proteins identified could be assigned to entries from the COG, KEGG, and KO databases, respectively. We first calculated the total protein intensity that corresponded to a specific function, by summing the intensities of all proteins associated with the function. Then, we estimated the percentage of each function, when the total intensities of all identified proteins were set to 100%.

**FIGURE 6:**
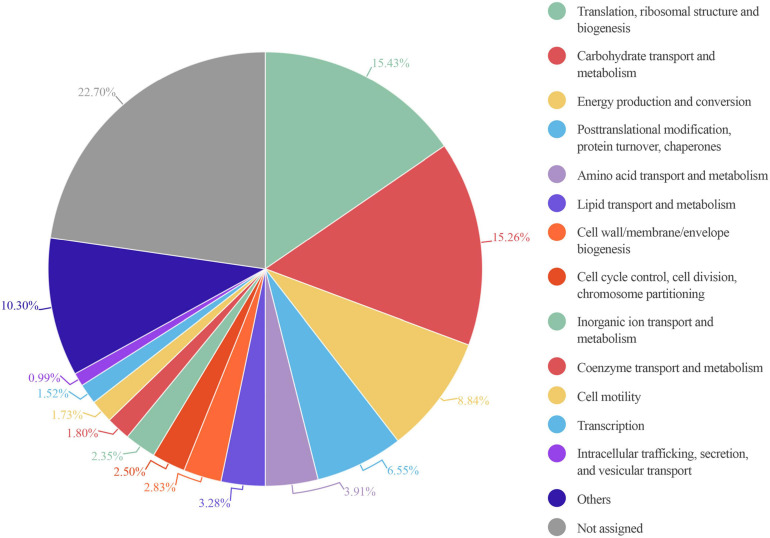
Circle plot representation of the abundance of COG functional entries displayed by the identified gut microbiota proteins from six healthy adults. Abundance in each sample is calculated as the percentage resulting from summing up all intensities belonging to proteins annotated to a specific COG category in this sample/total intensity of all proteins annotated to any COG category in this sample. The mean of the COG category abundance of the six samples is represented.

An enrichment analysis showed that the main core COG category functions in all 6 samples were: “translation, ribosomal structure and biogenesis,” “carbohydrate transport and metabolism,” and “energy production and conversion” consistent with previous studies (Debyser et al., 2016). These functions represented nearly 40% of the total protein intensity (Figure 6). This microbiota carbohydrate metabolism could facilitate the host exploitation of different food sources that would otherwise be indigestible. For example, S1 showed a high abundance of *Ruminococcus:* nearly 98% of all glycosidases identified in this sample belonged to members of this genus. This finding was consistent with the great capacity of *Ruminococcus* in carbohydrate degradation described previously (La Reau et al., 2016). We also found that other functions were highly represented in only some samples; for example, “cell motility” was highly represented in S5, and “coenzyme transport and metabolism” was highly represented in S3 (Supplementary Table 4).

The most abundant protein observed in our 6 samples was the glycolytic protein, glyceraldehyde-3-phosphate dehydrogenase (Supplementary Table 5). In addition, glutamate dehydrogenase, which was the most abundant intestinal protein in another study (Kolmeder et al., 2012), was in the top 30 proteins found in our samples.

**FIGURE 7:**
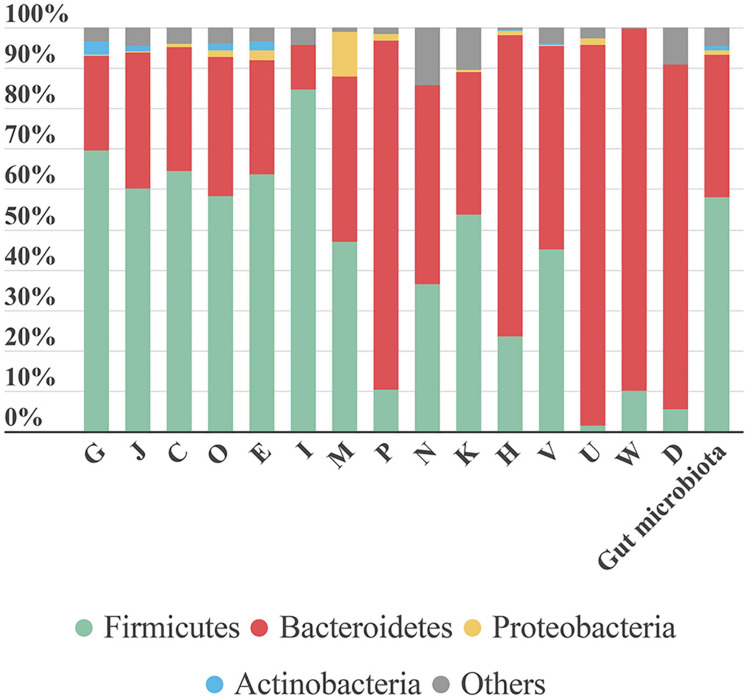
Bar plot representing the contribution of each phylum to the COG category analyzed. The last bar represents the taxonomic distribution of these phyla in the gut microbiota samples. Each phylum’s contribution in each sample is calculated as the sum intensity of proteins of this phylum annotated to the COG category. This bar plot represents the mean abundance of the six samples. G, Carbohydrate transport and metabolism; J, translation, ribosomal structure and biogenesis; C, energy production and conversion; O, post-translational modification, protein turnover, chaperones; E, amino acid transport and metabolism; I, lipid transport and metabolism; M, cell wall/membrane/envelope biogenesis; P, inorganic ion transport and metabolism; N, cell motility; K, transcription; H, coenzyme transport and metabolism; V, defense mechanisms; U, intracellular trafficking, secretion, and vesicular transport; W, extracellular structures; D, cell cycle control, cell division, chromosome partitioning.

From data obtained from MetaLab software we can match taxonomy with the functionality of each identified protein. Therefore, we could assign specific functions to specific taxa. As expected, a general analysis based on COG information, showed that proteins involved in the main functions belonged to the more abundant phyla. However, some specific functions were linked to a specific phylum (Figure 7). For example, “lipid transport and metabolism” was mostly represented by Firmicutes proteins. In contrast, functions like “inorganic ion transport and metabolism,” “coenzyme transport and metabolism,” “intracellular trafficking, secretion, and vesicular transport,” “extracellular structures,” and “cell cycle control, cell division, and chromosome partitioning” were mostly linked to Bacteroidetes. The high number of functions associated with Bacteroidetes could be due to its taxonomic diversity.

The function “inorganic ion transport and metabolism” was mostly represented by proteins involved in iron uptake. Iron is a key element in metabolism, and its availability influences the composition of gut microbiota (Yang et al., 2020). Therefore, iron uptake might confer an advantage to Bacteroidetes growth and survival.

A large proportion of the protein intensity (68–80%) associated with “intracellular trafficking, secretion and vesicular transport” was related to biopolymer transport (ExbB and ExbD). The ExbB and ExbD membrane proteins, along with a third one, form the TonB system that facilitates active transport of specific substances (in this case, biopolymers) across the membrane (Simon et al., 2007). In recent years, biopolymers have gained attention due to their applications in different industries and their role in bacterial pathogenicity. Biopolymers allow bacteria to grow under unfavorable conditions, because they can provide protection, energy storage, or biofilm components (Moradali and Rehm, 2020).

In the Proteobacteria phylum, most proteins were associated with the functional category “cell wall/membrane/envelope biogenesis.” We detected three types of proteins related to this category: (i) “opacity proteins and related surface antigens,” (ii) “outer membrane protein (porin),” and (iii) “outer membrane protein OmpA and related peptidoglycan-associated lipoproteins.” These are outer membrane proteins and have pathogenic functions; they act as virulence factors and are very immunogenic (Avidan et al., 2008). In S6, opacity proteins were associated with *Enterobacteriaceae*. Porins, which are associated with *Sutterella*, were identified in all samples. OmpA was highly represented in S3, where it comprised nearly 65% of the protein intensity associated with the “cell wall/membrane/envelope biogenesis” functional category and it was attributed to the species *B. wadsworthia*. When dysbiosis occurs, the Proteobacteria phylum tends to establish itself as the main phylum in the gut community, due in part to its broad adaptability. Further study on the role of Proteobacteria in different health stages might allow us to prevent its spread and avoid the associated health problems.

**FIGURE 8:**
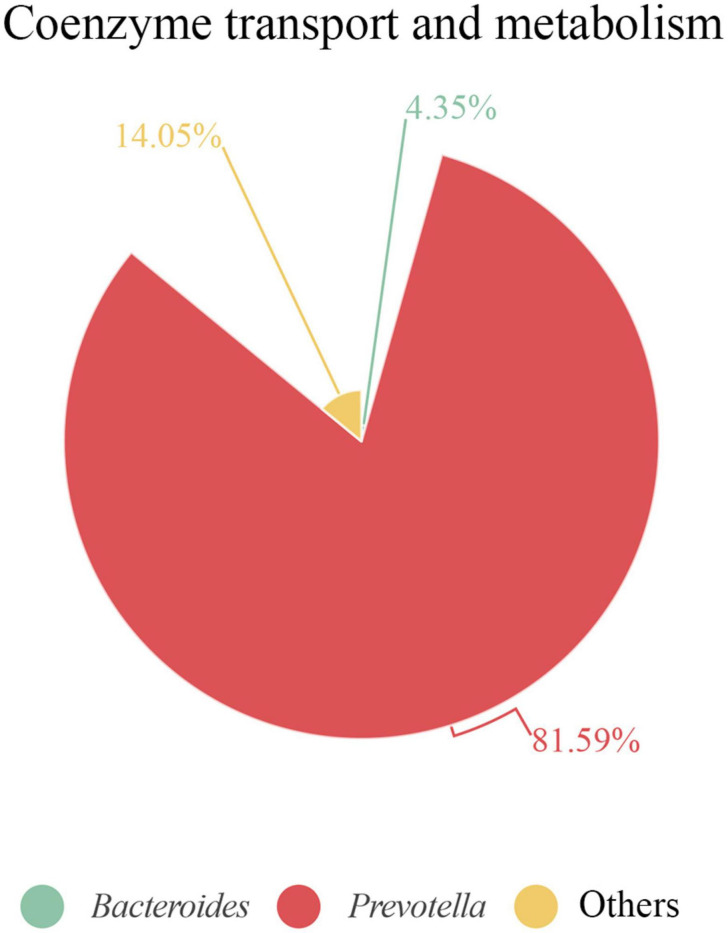
Proteins associated with coenzyme transport and metabolism in S3 at genus level. Contribution of each genus is represented as the percentage of protein intensity of the genus assigned to each COG category.

It is remarkable that, when analyzing each sample individually, we found that some functions were linked to only a few or even a single species. For example, in S3, we observed a strong link between proteins related to “coenzyme transport and metabolism” and the *Prevotella* genus. Of note, this function displayed a protein intensity 2–5 times greater in S3 than in the other samples (Supplementary Table 4). Indeed, over 80% of this COG category was represented by *Prevotella* proteins (Figure 8). The most abundant species in S3, was *P. copri*, which was the principal species related to this COG functional category. Moreover, 80% of the protein intensity associated to “coenzyme transport and metabolism” was related to cobalamin (vitamin B12) metabolism. The behavior of *P. copri* largely depends on the strain (De Filippis et al., 2019). Strains from Western individuals were associated with the synthesis of different B vitamins (De Filippis et al., 2019), consistent with our results. Most human gut taxa require B12, but most of these taxa lack *de novo* B12 synthesis. Therefore, most of these bacteria rely on cobalamin-uptake mechanisms to acquire sufficient B12 (Degnan et al., 2014a). It has been hypothesized that microbial communities might be manipulated to promote health by changing vitamin intake, due to the high competition for cobalamin (Degnan et al., 2014b). Members of Firmicutes and Actinobacteria phyla harbor complete B12 biosynthetic pathways (Rowley and Kendall, 2019). In contrast, Bacteroidetes, which lacks *de novo* B12 biosynthetic genes, encodes several cobalamin transporters (Degnan et al., 2014a), which could be detected among the identified proteins. Interestingly, the cobalamin biosynthesis protein, Cbik (COG4822), which is one of the first proteins that participates in *de novo* cobalamin synthesis, was related to Firmicutes. In contrast, an outer membrane cobalamin receptor protein (COG4206) was associated with *Prevotella*. Additionally, some proteins required for the activation of B12 synthesis were associated with *Bacteroides* (e.g., cobalamin biosynthesis protein CobN; COG1429) and *Prevotella* (e.g., cobalamin adenosyltransferase; COG2096). Furthermore, vitamin B12 consumption has been related to an increase in the relative abundance of *Prevotella* over *Bacteroides* (Carrothers et al., 2015), which might potentially explain the high abundance of the *Prevotella* genus observed in S3.

This example is consistent with the premise that in each individual different biological functions are linked to specific taxa that contribute to specific host-microbiota interactions. This principle should be kept in mind, particularly in the field of personalized medicine and nutrition (Mills et al., 2019).

Another important function of the gut microbiota in human physiology is the synthesis of short chain fatty acids (SCFAs) through the fermentation of different non-digestible carbohydrates. SCFAs are recognized by G-coupled receptors that trigger the secretion of intestinal peptides (Cani, 2018). The main SCFAs are butyrate, propionate, and acetate. Each of these SCFAs plays important roles in human health, such as regulating the epithelial barrier integrity, providing anti-inflammatory effects, and serving as the primary nutrient for colonocytes (Morrison and Preston, 2016; Yamamura et al., 2020). Butyrate is the most important energy source for colonocytes. Moreover, it influences the microbial environment and ecology, and it prevents the expansion of pathogens (Cani, 2018). Due to current interest in the beneficial roles that SCFAs perform in the intestine (Riviere et al., 2016), we analyzed SCFA production by Firmicutes in all 6 samples. Nineteen KOs were associated with the KEGG pathway involved in “butanoate metabolism” (Supplementary Table 6). Firmicutes proteins represented 62% of the total butyrate pathway protein intensity, and the main genus was *Faecalibacterium* (a member of *Ruminococcaceae*), which comprised 25% of the intensity. These results were consistent with previous studies that showed that members of the Firmicutes phylum were the main butyrate producers, which highlighted the role of *Ruminococcaceae* family in butyrate production (Louis et al., 2009; Morrison and Preston, 2016; Yamamura et al., 2020). The KOs identified in butyrate production from acetyl-CoA were all assigned to the Firmicutes phylum. The KOs related to SCFA production that were assigned to Bacteroidetes were not directly involved in the biosynthetic pathway, instead, they were related to acetyl-CoA production from pyruvate and the interconversion of succinate-fumarate (Supplementary Figure 3).

#### Human Proteins Detected in Proteomic Studies of Stool Samples

The MetaLab program allows the identification of human proteins that are present in stool samples. We identified up to 92 human proteins throughout the 6 human samples. Interestingly, although human peptides only accounted for 2% of the total identified peptides, the intensities of these peptides represented up to 13% of the total peptide intensity in S1. This difference between the abundance of identified peptides and their intensity was consistent with findings in a previous study (Zhang et al., 2017). The most abundant proteins were chymotrypsin C, trypsin 2, phospholipase A2, and alpha amylase. Alpha amylase, a starch degradation protein, is produced by two genes, in saliva and mammary gland or in the pancreas that is secreted into the duodenum in digestive juices (Groot et al., 1989).

**FIGURE 9:**
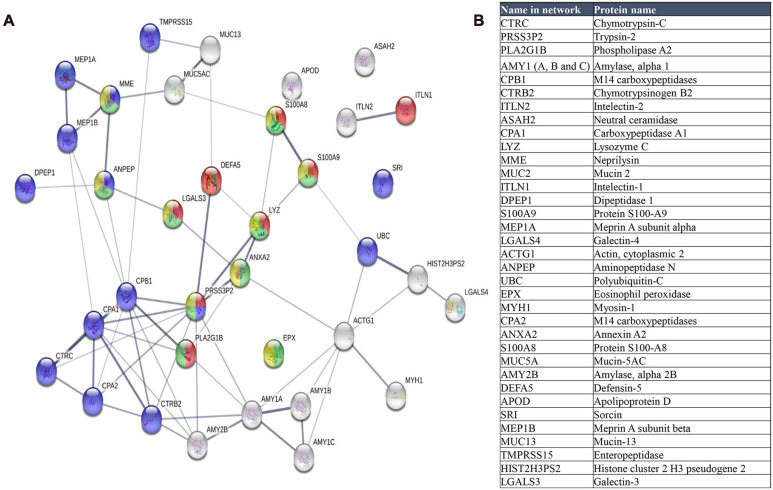
**(A)** STRING map protein-protein interaction network of the 35 more abundant human proteins identified in this study. Line thickness indicates the strength of data support. The colors represent different processes; red: antimicrobial humoral response, green: neutrophil mediated immunity, blue: proteolysis and yellow: neutrophil degranulation. **(B)** Table of these proteins ordered according to decreasing abundance.

A group of 35 human proteins that were found in all stool samples at high abundance (Figure 9 and Supplementary Table 7) were selected for STRING analysis. These proteins represented over 80% of the total intensity of identified human proteins. Most of these selected proteins were also found in previous studies on the microbiome (Zhang et al., 2017; Zhong et al., 2019), including intestinal mucin proteins (MUC2, MUC5B, and MUC13) and digestive enzymes, like chymotrypsinogen B2 and carboxypeptidases. MUC2 is the predominant mucin type in the stomach mucus layer. Intestinal mucus provides a protective, lubricating barrier against particles and infectious agents, and it interacts with microorganisms. For example, microbiota can feed on mucin glycans and convert them into SCFAs that supply colonocytes and other gut epithelial cells with energy (Ouwerkerk et al., 2013). Interestingly, MUC13 is abundant in many adenocarcinomas (Sheng et al., 2019), where it serves as a potential prognostic factor (Filippou et al., 2018).

When we analyzed the selected 35 human proteins with the STRING program (version 11.0), only one protein (Mucin 2) did not match in the STRING database. The other proteins showed significant protein-protein interactions (*p* = 1.0 × 10^–16^, Figure 9). The antimicrobial humoral response was the most significant enrichment process (*p* = 3.10 × 10^–7^), followed by proteolysis (*p* = 7.32 × 10^–6^), and immune system-related processes, including neutrophil-mediated immunity (*p* = 7.32 × 10^–6^), neutrophil degranulation (*p* = 3.35 × 10^–5^), granulocyte migration (*p* = 3.4 × 10^–5^), and immune effector process (*p* = 4.84 × 10^–5^).

We found that the enrichment of the antimicrobial humoral response process was due to the high number of antimicrobial peptides identified in the samples, consistent with other metaproteomics studies (Zhong et al., 2019). These antimicrobial peptides or proteins, like defensin-5, lysozyme c, and phospholipase A2, accomplish important roles in the defense against bacteria (Zhong et al., 2019). Apart from antimicrobial peptides, we identified other proteins like Interlectin 1, S100A9, S100A8, and galectin 3, which are related to the antimicrobial humoral response. Interlectin 1 is a lectin that binds microbial glycans and is used by the immune system to discriminate human cells from microbes (Wesener et al., 2015). S100A9 and S100A8 can induce neutrophil chemotaxis and adhesion, and they are found as a complex (S100A8/A9) called calprotectin. High levels of calprotectin have been observed in inflammatory bowel disease and other gastrointestinal disorders (Lehmann et al., 2019), and thus, it was proposed as a marker (Fukunaga et al., 2018). Galectin 3 is a lectin involved in neutrophil activation and adhesion. Galectin 3 plays an important role in inflammation and is associated with several diseases (Sciacchitano et al., 2018).

Proteolysis was also highly represented in the STRING results. Proteases play important roles in gastrointestinal disorders (Antalis et al., 2007). Enteropeptidase is responsible for activating the conversion of pancreatic trypsinogen to trypsin, which activates other proenzymes (e.g., chymotrypsinogen, procarboxypeptidases, and others). Trypsin is also involved in processing defensins. The most significantly enriched functions were metallopeptidase activity, hydrolase activity, and peptidase activity (*p* = 5.23 × 10^–9^). We identified the metalloprotease, meprin A (alpha and beta subunits), which is implicated in inflammation and tissue remodeling (Kaushal et al., 2013).

We also observed a cellular component enrichment. Proteins related to the extracellular space (*p* = 3.59 × 10^–19^) and extracellular regions (*p* = 5.40 × 10^–18^) were the most significantly enriched. This finding was consistent with the fact that these extracellular proteins might have been dragged through the human intestine during fecal sampling.

## Concluding Remarks

In this study, we designed and evaluated different SSP methods for the metaproteomic study of gut microbiota. We found that a 45-min processing of the fecal sample prior to protein extraction was critical for good protein recovery and peptide identification. Furthermore, the cell breaking method determined the number of peptides that could be identified and, more interestingly, the particular taxa that would be enriched in the metaproteomic taxonomic profile. A sonication procedure prior to bead-beating in microbial cell disruption raised the numbers of proteins and peptides identified. However, bead-beating alone increased the number of Proteobacteria proteins identified. This method could be more informative for studies related to diseases, because Proteobacteria phylum was associated with different dysbiosis stages, which facilitated the differentiation of healthy and unhealthy stages. These results are relevant to future metaproteomic studies on the human gut microbiota, because they inform the selection of the best protocol, based on the specific interest in a particular taxon-related disease.

The metaproteomic studies enabled the profiling of protein functions in the microbiota from 6 healthy individuals. We found interesting correlations between specific microbial functions relevant to the host and the main taxa involved. For example, vitamin B12 process was mainly produced by proteins in the *Prevotella* genus. This could be particularly interesting when searching for links between certain taxon-related proteins and specific beneficial or detrimental host stages in future health-disease metaproteomic studies. Finally, we also detected 92 human proteins, which were mostly of them related to the antimicrobial humoral response.

The newly described protocols for enriching specific taxa can facilitate the functional analysis of both microbial and human proteins in the human gut. This information can improve the design of future metaproteomic studies on gut microbiota and open up new prospects in the field of host–pathogen interactions in different diseases.

## Data Availability Statement

The datasets presented in this study can be found in online repositories. The names of the repository/repositories and accession number(s) can be found below: https://www.ebi.ac.uk/pride/archive/, PXD020786 and PXD025659.

## Ethics Statement

Ethical review and approval was not required for the study on human participants in accordance with the local legislation and institutional requirements. The patients/participants provided their written informed consent to participate in this study.

## Author Contributions

CG-D, RM-L, and IZ performed the experiments. CG-D analyzed, interpreted the data, and wrote the manuscript. RM-L and AP contributed to the data analysis and wrote the manuscript. AP carried out the statistical analysis. EP and ER supported the bioinformatic analysis. MH designed and supervised the proteomic experiments. JA participated in the data analyses and critically reviewed the manuscript. CG and LM conceived and designed the experiments, supervised the experimental work, and critically reviewed the manuscript. All the authors approved the final version of the manuscript.

## Conflict of Interest

The authors declare that the research was conducted in the absence of any commercial or financial relationships that could be construed as a potential conflict of interest.

## Footnotes

1 http://www.proteomexchange.org

2 https://db.cngb.org/microbiome/genecatalog/genecatalog_human/

3 www.uniprot.org

4 http://shiny.imetalab.ca/

5 http://www.kegg.jp

6 https://infogram.com/

## Supplementary Material

Supplementary Figure 1 Taxonomic distribution at superkingdom level of the gut microbiota from stool sample H1 displayed by each one of the three replicates carried out with the different protocols tested (PA–PF). The intensity of each superkingdom (directly related to its abundance) is represented as the sum of the intensities of all the distinctive peptides assigned to that superkingdom. The intensity corresponding to human peptides was represented separately from microbial Eukaryota.

Supplementary Figure 2 Sum of the intensity of all distinctive peptides of each taxonomic level in each protocol (PA–PF). ^∗∗^*p* < 0.0098; ^∗^*p* < 0.037.

Supplementary Figure 3 Butyrate metabolism KEGG pathway downloaded from KEGG website (http://www.kegg.jp). Colored KO numbers indicate proteins identified in this study. Firmicutes proteins are colored in blue and Bacteroidetes proteins in purple.

Supplementary Table 1 Metrics of metaproteome analysis of H1 using protocols A–F.

Supplementary Table 2 Functional analysis of the proteomes obtained with different protocols. The abundance of each COG category is represented as the sum of the intensities of all proteins annotated to that COG category in each sample.

Supplementary Table 3 Taxonomy of human gut microbiota of six healthy adult individuals.

Supplementary Table 4 Functional analysis of the proteomes from the human gut microbiota of six healthy adults. The abundance of each COG category is represented as the sum of the intensities of all the proteins annotated to that COG category in each sample. The box color indicates a gradient of intensity. The intensity marked in red is of special interest and is discussed in depth in the text.

Supplementary Table 5 The thirty most abundant microbial proteins identified in the study.

Supplementary Table 6 KOs entries belonging to proteins from butanoate metabolism pathway identified in the study.

Supplementary Table 7 List of the most abundant human proteins identified in the six human gut microbiota samples used for the STRING analysis.
