# Supplementary material for: Distinct Human Gut Microbial Taxonomic Signatures Uncovered With Different Sample Processing and Microbial Cell Disruption Methods for Metaproteomic Analysis
Source: Front Microbiol. 2021 Jul 5;12:618566. doi: 10.3389/fmicb.2021.618566 (PMC8287257; doi:10.3389/fmicb.2021.618566)
Supplement: Supplementary Figure 1 — Taxonomic distribution at superkingdom level of the gut microbiota from stool sample H1 displayed by each one of the three replicates carried out with the different protocols tested (PA–PF). The intensity of each superkingdom (directly related to its abundance) is represented as the sum of the intensities of all the distinctive peptides assigned to that superkingdom. The intensity corresponding to human peptides was represented separately from microbial Eukaryota. [file Data_Sheet_1.docx]

***Supplementary Material***

**
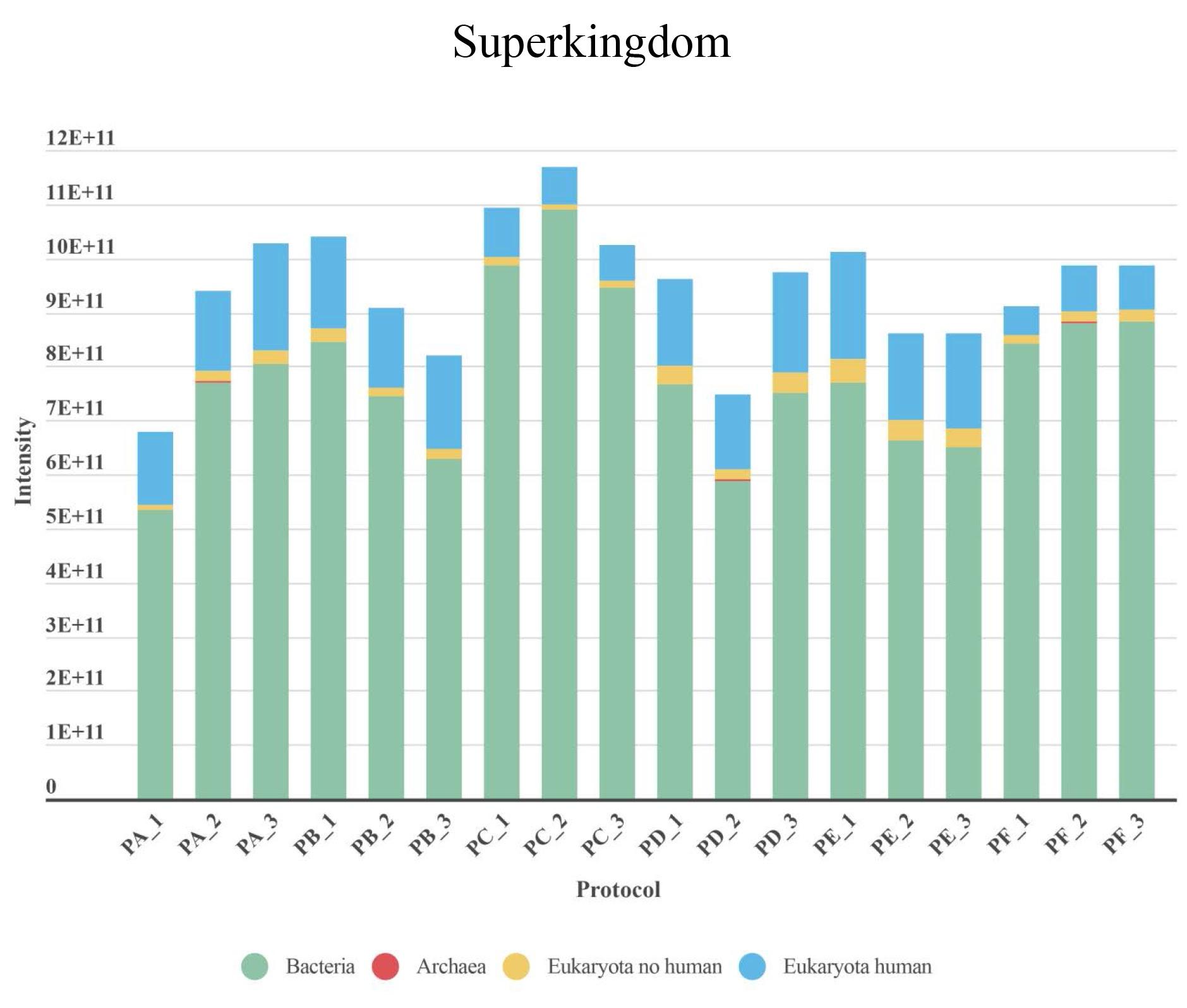
**

**Supplementary Figure 1.** Taxonomic distribution at superkingdom level of the gut microbiota from stool sample H1 displayed by each one of the three replicates carried out with the different protocols tested (PA-PF). The intensity of each superkingdom (directly related to its abundance) is represented as the sum of the intensities of all the distinctive peptides assigned to that superkingdom. The intensity corresponding to human peptides was represented separately from microbial Eukaryota.


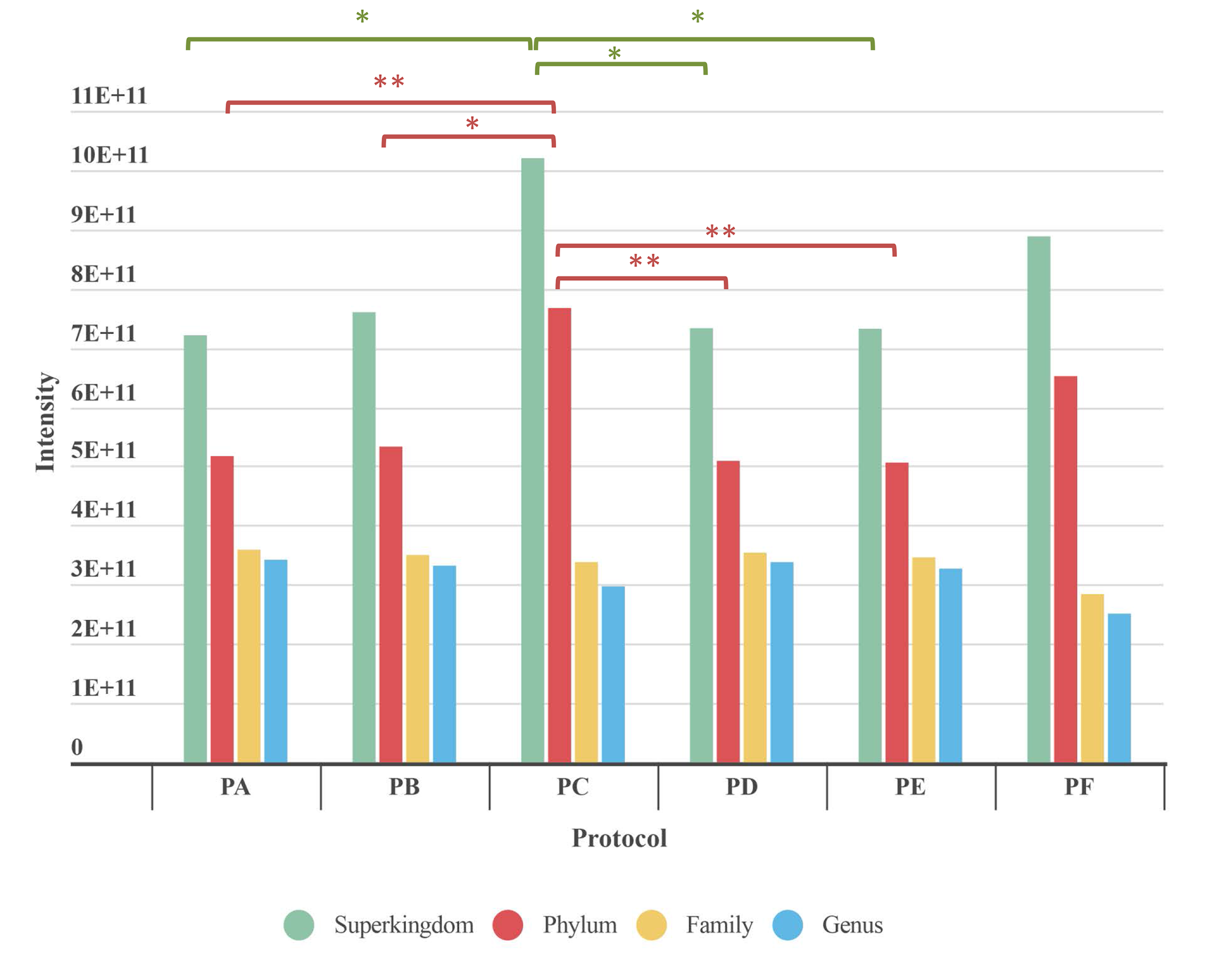


**Supplementary Figure 2.** Sum of the intensity of all distinctive peptides of each taxonomic level in each protocol (PA-PF). ***p <*0.0098. **p <*0.037.


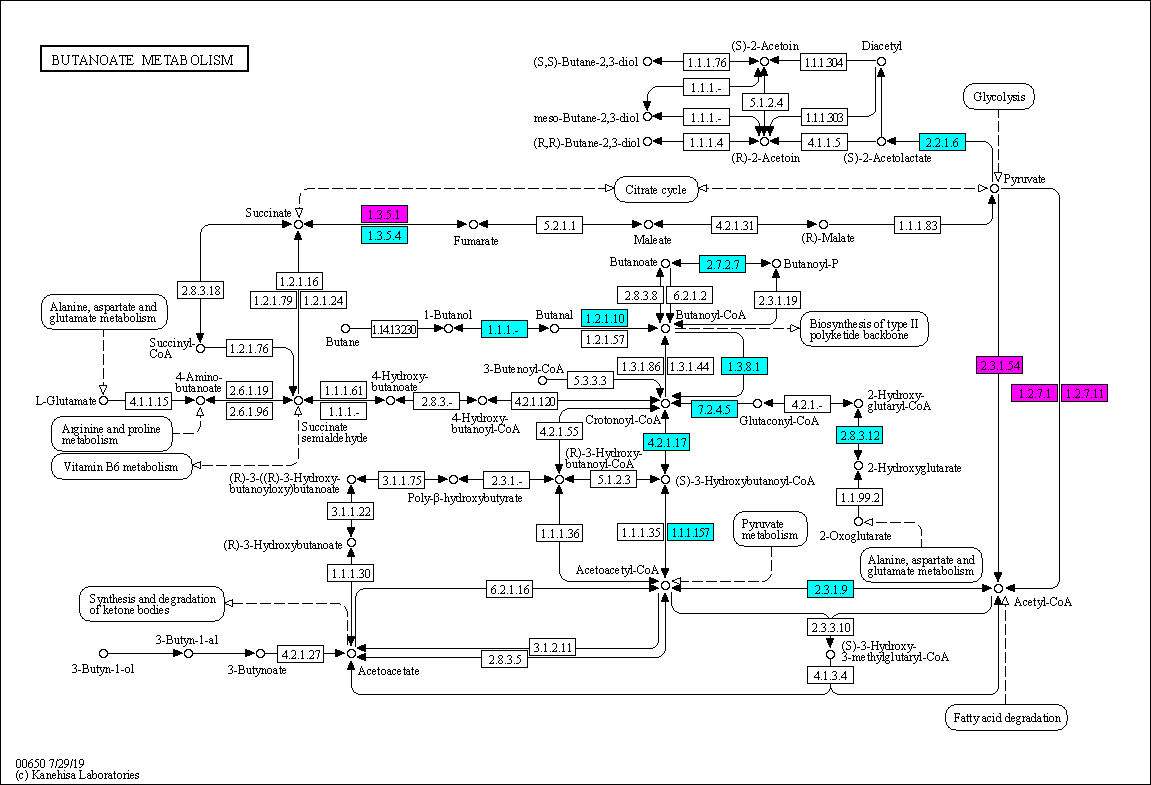


**Supplementary Figure 3.** Butyrate metabolism KEGG pathway downloaded from KEGG website (<http://www.kegg.jp>). Colored KO numbers indicate proteins identified in this study. Firmicutes proteins are colored in blue and bacteroidetes proteins in purple.
